# Editorial: Inhibitory function in auditory processing

**DOI:** 10.3389/fncir.2015.00045

**Published:** 2015-09-01

**Authors:** R. M. Burger, Ian D. Forsythe, Conny Kopp-Scheinpflug

**Affiliations:** ^1^Department of Biological Sciences, Lehigh UniversityBethlehem, PA, USA; ^2^Department of Cell Physiology and Pharmacology, College of Medicine, Biological Sciences, Psychology, University of LeicesterLeicester, UK; ^3^Division of Neurobiology, Department of Biology II, Ludwig-Maximilians-University MunichPlanegg-Martinsried, Germany

**Keywords:** GABA, glycine, nitric oxide, plasticity, gap junctions, sound localization, MNTB, co-release

In recent decades, with the convergence of high-resolution anatomical and physiological techniques, a perspective is emerging on inhibition in the nervous system that recognizes the vast diversity of functions it serves. These include roles in modulation, development, and plasticity, in addition to the common perception of inhibition as spike suppression. Progress toward this more nuanced understanding of inhibition has derived from many studies across the nervous system, but here we focus on part of the brainstem auditory system, where discrete inhibitory nuclei interact in unique and fascinating ways to integrate and compute binaural information in the circuitry for sound source localization.

We have solicited studies for this special topic on inhibition in the auditory brainstem circuitry from laboratories around the world. The assembled manuscripts provide an authoritative collection of concepts across the breadth of neuroscience research on inhibitory function that focus on three major themes.

## Inhibition in the superior olive: The medial nucleus of the trapezoid body (MNTB)

A major advantage of investigating inhibition in the auditory pathway is the distribution of inhibitory centers among its subthalamic nuclei. In general, these nuclei are involved in computing the azimuth location of a sound source, by integrating the acoustic stimulus from both ears. The superior olivary complex (SOC) is the first region of the brain to compute sound location by comparing the input to the two ears. The MNTB is central to this circuitry and is highly specialized; being driven by the Calyx of Held (one of the largest synapses in vertebrates) and providing a powerful glycinergic inhibition to its targets. Despite its pivotal role in this circuit and extensive investigation, our understanding remains incomplete, the details of its inputs, output, and sound encoding are still being explored.

The MNTB projects to multiple targets in the SOC including those that process cues for sound localization. Two studies investigate what information is conveyed by these neurons, with regard to both temporal and spectral encoding. The first, by Koka and Tollin (2014) demonstrates that the MNTB accurately and linearly encodes spectral information. The spectral content represented in output spiking is crucial for understanding what binaural comparisons the MNTB's targets can make. For example the medial superior olive (MSO), which receives contralateral ear-derived inhibition from the MNTB and which also receives an analogous inhibitory input from the ipsilateral ear via the LNTB. In an innovative *in vitro* study, Roberts et al. (2014) compared these two neighboring inhibitory inputs to the MSO. They demonstrated that the two inputs have similar latencies but do not share identical temporal encoding properties.

The MNTB also receives inhibition, the origin of which has been a source of speculation for many years. Two studies help refine our understanding of inhibitory input to the MNTB. First, Albrecht et al. (2014) identify the ventral nucleus of the trapezoid body (VNTB) as a major source of glycinergic input to the MNTB. They also show that this input follows a similar developmental pattern to that of the MNTB itself, with mixed GABA/glycine release early in development followed by primarily glycine release later in development. Second, Trojanova et al. (2014) show that one target of this glycinergic input is targeting the presynaptic terminals of the *glutamatergic* Calyx of Held. This study shows a compelling pattern of glycine receptor expression on the terminals at locations neighboring putative glutamate release sites and apposed to inhibitory terminals. This study shows anatomical evidence suggesting that glycine receptors are poised to modulate release of excitatory transmitter directly via spillover of inhibitory transmitter.

The MNTB appears to be so central to auditory circuitry, that it is difficult to imagine how the network could adapt to its absence, but genetic tools have allowed Altieri et al. (2014) to address this question. They investigated the development of markers for inhibition in the SOC in *Engrailed*^−∕−^ mice that fail to develop their MNTB nucleus. These mice are thus deprived of a major source of glycinergic inhibition to the LSO, MSO, and superior paraolivary nucleus (SPN). Surprisingly, development of immunohistochemical markers for glycinergic transmission, although delayed, reach typical levels in adulthood, demonstrating remarkable developmental plasticity in this system and provide evidence for alternative sources of inhibitory input.

## Short-term, long-term, and novel mechanisms of inhibitory plasticity

Synaptic plasticity allows developmental change and activity-dependent adaptation of information transmission throughout the nervous system. In the auditory pathway, myriad examples of plasticity of inhibitory signaling are demonstrated. They include classical forms such as LTP, LTD, depression and facilitation, as well as novel forms described below.

Plasticity is a prominent feature of processing in another region of the auditory brainstem, the dorsal cochlear nucleus (DCN), which includes complex intra-CN inhibitory circuitry driven by the auditory nerve via the tuberculoventral interneuron. Sedlacek and Brenowitz (2014) carefully dissects this circuit to reveal how different contributions of short term synaptic plasticity among direct and disynaptic pathways in the DCN strongly influence its primary output neuron, the fusiform cells. Indeed, the circuitry of the fusiform cell is complex and involves several cell types intrinsic to the DCN. Apostolides and Trussell (2014) explores a poorly understood component of this circuitry, called the superficial stellate cell (SSC). SSCs not only form inhibitory synapses but also are electrically coupled to fusiform cells as well as one another. SSCs appear well positioned to mediate a coordinated non-auditory derived modulation of DCN output. In the SOC, Kramer et al. (2014) investigated short-term synaptic plasticity using a novel “marathon” stimulation protocol to reveal components of synaptic plasticity rarely analyzed in previous works. They demonstrate that the inhibition to the LSO via MNTB is very robust with respect to reliability, but more prone to depression than previously reported in studies using less demanding stimuli.

Long-term synaptic plasticity is a hallmark of excitatory synaptic coupling, particularly during development. In contrast, long-term plasticity at inhibitory synapses is less commonly studied. One exception is at the MNTB-LSO synapse where Kotak and Sanes (2014) have previously demonstrated GABA_B_ receptor-dependent long term depression, especially early in development. In their current paper, they add to this body of work by demonstrating that this synapse also expresses long-term potentiation, but somewhat later in development. This potentiation surprisingly also depends on GABA_B_ receptor function.

Plasticity of inhibition can also occur indirectly, as demonstrated in the SPN, a synaptic target of the MNTB. Yassin et al. (2014) in a comparative study across species and between the SOC nuclei reveal that nitric oxide (NO) signaling dynamically modulates inhibitory strength. Interestingly, NO acts postsynaptically through a cGMP dependent pathway to suppress KCC2. This outwardly directed potassium chloride co-transporter is crucially involved in setting the neuronal Cl^−^ reversal potential. The NO-dependent depolarizing shift in reversal potential demonstrates a possible means to modulate inhibition in SPN neurons, without influencing inhibition in other collateral targets of the MNTB.

## Diversity of inhibition in monaural and binaural nuclei

Inhibitory neurons of the SOC typically release two or more transmitters in early development, but revert to a single dominant transmitter following hearing onset. This general principle has been refined in the last two decades. Recently however, it has become apparent that transmitter release in *mature* auditory circuitry may be more complex than previously appreciated. In this issue, Case et al. (2014) extends these studies to investigate the role of vesicular glutamate transporter expression in “glycinergic” MNTB neurons. Two other studies, by Fischl and Burger (2014) and Nerlich et al. (2014) extend recent findings that GABAergic and glycinergic inputs to the cochlear nucleus are dominated by a single neurotransmitter at low stimulus rates, but surprisingly become multi-transmitter release synapses at high stimulus rates. This principle applies to both birds and mammals and at multiple synapses. In a complementary study Xie and Manis (2014) use optical tools to finely dissect the properties of both GABAergic and glycinergic transmission in two types of cochlear nucleus neurons, showing that kinetics and short-term plasticity are heavily dependent on the synaptic target.

One underappreciated aspect of inhibition that is emerging in the literature is that classical synaptic inhibition can engage voltage gated ion channels and signaling pathways beyond their classical receptors. Hamlet et al. (2014) investigate functional coupling between GABA receptors and low voltage gated potassium channels in nucleus laminaris (NL) of the chick. In NL, GABA is depolarizing and activates this potassium current. The present study demonstrates the precise and profound interplay between the Cl^−^ and K^+^ conductances occurring in both pre and postsynaptic compartments of this circuitry.

## A broader view of inhibition

Finally, this Research Topic presents two review offerings. The first, from Ohmori (2014) focuses on specializations of the sound localization pathway in the chick. This is a major model system that shares many features with mammalian circuitry. The second from Grothe and Pecka (2014) presents a novel hypothesis concerning the evolutionary origins of the role of inhibition in the superior olive by synthesizing what is known about the origin of the tympanic ear, the fossil record, and inhibitory circuitry in extant animals.

Together these studies and perspectives provide a taste of current concepts, with promises of more exciting insights into auditory function around the corner. Inhibition is nonetheless important for its suppression of mere excitation, and as we see here this field is vibrant and forward-looking. We hope that neuroscientists investigating the physiology of inhibition beyond the auditory system will find this work equally exciting and we thank all of our contributing authors for their excellent work. And to you, our readers, we hope you find some inspiration for your own research.

### Conflict of interest statement

The authors declare that the research was conducted in the absence of any commercial or financial relationships that could be construed as a potential conflict of interest.
